# Characterization of Temperature and Humidity Dependence in Soft Elastomer Behavior

**DOI:** 10.1089/soro.2023.0004

**Published:** 2024-02-13

**Authors:** Elze Porte, Sophia Eristoff, Anjali Agrawala, Rebecca Kramer-Bottiglio

**Affiliations:** ^1^ Department of Mechanical Engineering and Materials Science, Yale University, New Haven, Connecticut, USA.; ^2^ Department of Mechanical Engineering, University College London, London, United Kingdom.; ^3^ Department of Civil, Environmental & Geomatic Engineering, University College London, London, United Kingdom.

**Keywords:** soft robotics, sensors, space, environmental conditions, tensile test, electromechanical test

## Abstract

Soft robots are predicted to operate well in unstructured environments due to their resilience to impacts, embodied intelligence, and potential ability to adapt to uncertain circumstances. Soft robots are of further interest for space and extraterrestrial missions, owing to their lightweight and compressible construction. Most soft robots in the literature to-date are made of elastomer bodies. However, limited data are available on the material characteristics of commonly used elastomers in extreme environments. In this study, we characterize four commonly used elastomers in the soft robotics literature—EcoFlex 00-30, Dragon Skin 10, Smooth-Sil 950, and Sylgard 184—in a temperature range of −40°C to 80°C and humidity range of 5–95% RH. We perform pull-to-failure, stiffness, and stress-relaxation tests. Furthermore, we perform a case study on soft elastomers used in stretchable capacitive sensors to evaluate the implications of the constituent material behavior on component performance. We find that all elastomers show temperature-dependent behavior, with typical stiffening of the material and a lower strain at failure with increasing temperature. The stress-relaxation response to temperature depends on the type of elastomer. Limited material effects are observed in response to different humidity conditions. The mechanical properties of the capacitive sensors are only dependent on temperature, but the measured capacitance shows changes related to both humidity and temperature changes, indicating that component-specific properties need to be considered in tandem with the mechanical design. This study provides essential insights into elastomer behavior for the design and successful operation of soft robots in varied environmental conditions.

## Introduction

Robots are used to explore dangerous and difficult-to-access environments, such as the Mars Rover in space or autonomous underwater vehicles in the deep-sea. Soft robots can offer additional advantages because of their potential to adapt to uncertain environments^1–5^ and to pack into tight spaces.^6^ Real environments pose different conditions to the robots than the laboratory environments in which they are developed and tested. For example, temperatures on the Moon's equator can vary between 123°C during the day and −170°C at night, and the temperature can drop below −230°C at the poles.^7,8^ Extreme low humidity conditions can be experienced on the Earth toward the poles and extreme high humidity in hot climates toward the equator, since the ability to hold water vapor is temperature dependent.^9^ Successfully operating soft robots in any of these environments requires a detailed understanding of the response to the environmental conditions of their constituent materials.

The base materials in soft robots' comprising structures, actuators,^10–13^ and sensors^14–17^ are often commercial silicones. These materials are attractive to use because they can often stretch to very large strains (>50%) and are easy to process. The material characteristics provided by the manufacturer, such as Shore A hardness and tensile strength, are not always sufficient to accurately predict the behavior of the robot, which is why more detailed characterizations have been carried out in recent studies to advance soft robot design.^18–20^ Such studies highlight the nonlinear and viscoelastic behavior of the silicones, which is not captured by the manufacturer's data. The detailed characterizations can help in soft robot design, since the nonlinear material parameters can be used in finite element (FE) models and help better predict the robot's response across a range of motions.

Successful operation of soft robots in various environments will require a further understanding of the constituent materials in appropriate conditions. To date, limited information is available on the response of commonly used elastomers in soft robots to environmental conditions, including temperature and humidity. Although the curing temperature of polydimethylsiloxanes (PDMS) is known to be an important factor for the properties of the material,^21,22^ these studies often do not include the effect of the operating temperature. A study on EcoFlex 00-30 found an increase in stiffness with increasing temperature of the environment for a temperature range of −40°C to 140°C,^23^ indicating that environmental temperature should be considered in the operational model of the robots.

Elastomers are known to swell when exposed to water or solvents,^24^ but research mostly focuses on achieving large volumetric expansion in elastomeric seals^25,26^ and provides little insight into the effects of humidity on the broader mechanical behavior of the elastomers required for soft robot operation.

Recent work has focused on building a database with the mechanical behavior of commercially available silicones to provide the soft robotics community with modeling parameters to run FE simulations and to choose appropriate materials for their applications.^19^ In line with this effort, we are characterizing four common commercial elastomers (EcoFlex 00-30, Dragon Skin 10, Smooth-Sil 950, and Sylgard 184) for a range of temperature and humidity conditions. We provide a case study on the performance of elastomer-based capacitive sensors to showcase environmental effects on a soft robotic component. The aim of this work is to provide insight into the effect of environmental conditions on the material characteristics of elastomers to aid the design of soft robots for both terrestrial and extraterrestrial exploratory missions, with a special focus on working toward lunar missions.

## Materials and Methods

### Elastomer sample preparation

**FIG. 1.**
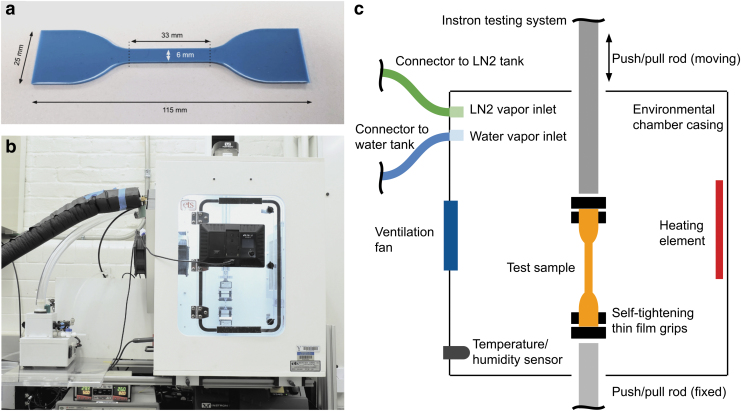
Overview of the testing setup: **(a)** example of Smooth-Sil 950 sample; **(b)** picture of the environmental chamber setup; **(c)** schematic of the environmental chamber and material testing system.

Samples were prepared according to ASTM D412 using dumbbell shape C and cast in acrylic molds with a thickness of 2 mm. Figure 1a shows an example of a Smooth-Sil 950 sample. For the failure tests, the size of the dumbbell shape was reduced by 50% to ensure tests could be performed within the boundaries of the environmental chamber (38 × 46 × 61 cm). This sample size is not part of the ASTM D412, but was an unavoidable alteration due to the setup restrictions. Dragon Skin 10 Medium (Smooth-on, Inc.) and EcoFlex 00-30 (Smooth-on, Inc.) were prepared by mixing parts A and B in a 1:1 ratio. Smooth-Sil 950 (Smooth-on, Inc.) and Sylgard 184 (Dow) were prepared by mixing parts A and B in a 10:1 ratio.

All materials were mixed in a centrifugal mixer (Thinky ARE-310) at 2000 rpm for 30 s and degassed at 2200 rpm for 30 s. The elastomers were poured in the molds and put in a vacuum chamber (−80 to −100 kPa) to remove air bubbles from the mixtures. Excess elastomer was removed with a flat metal scraper to level the samples. All samples were cured at room temperature. Three equally spaced dots [3% wt. black Silc Pig, 97% EcoFlex 00-10 (1:1, A:B), Smooth-on, Inc.] were painted on the narrow middle to image the real strain. The width of the samples was assumed to be the casted width (6 mm). The thickness of the samples was found by measuring the thickness with a micrometer at three different locations on the sample and taking an average.

### Sensor manufacturing

Two sets of sensors were prepared: one with Dragon Skin 10 and one with Smooth-Sil 950 as base material. Capacitive sensors were manufactured similar to the method described in an earlier work.^14,17^ In short, expanded intercalated graphite (EIG) was prepared as conductive filler for the sensor electrodes. Expandable graphite (5 g; Sigma Aldrich) was expanded at 800°C and soaked in cyclohexane (0.5 L; Thermo Fisher Scientific). The EIG/cyclohexane mixture was sonicated for 1 h (70%; Q500 1/2” tip, Qsonica) and sieved through a 212 μm sieve. After settling of the particles (1 h), excess cyclohexane was decanted and the mixture was boiled down to ∼3% wt. EIG. The EIG mixture was manually stirred into the elastomers at ∼79% wt. to achieve 10% wt. EIG in the electrodes after evaporation of the cyclohexane during curing. Elastomers were prepared using the same mixing ratios as for the elastomer sample preparation.

The sensors consist of five layers; three electrode layers and two dielectric layers. The layers of the sensors were coated using a film applicator (SH0340; TQC Sheen). The applicator was set to 500 μm for all electrode layers and to 1200 μm for the Dragon Skin dielectric and 800 μm for the Smooth-Sil 950 dielectric layers. The final thickness of the cured layers was slightly lower than the coated thickness due to spreading of the elastomers before curing.

The initial electrode and first dielectric layer were coated with a width of 110 mm. The second electrode layer was coated on top of the dielectric layer to a width of ∼100 mm, by covering one edge with a paper mask. The second dielectric layer was coated to a width of ∼100 mm by covering the other edge with a paper mask. The final electrode layer was coated on top of the second dielectric layer to approximately the same width, again using a paper mask.

Sensors were cut to size (110 × 10 mm) with a laser cutter (VLS 2.30; Universal Laser Systems). The first and third electrode were connected at the short edge by painting the edge with a conductive glue made from SilPoxy (Smooth-On, Inc.) and EIG with the same mixing ratio as the electrodes. Copper strips were attached to the first and second electrode using the same conductive glue. The entire sensor was encapsulated in a thin layer of elastomer to prevent the electrodes from shorting in the humidity tests. A schematic of the different sensor layers and a picture of a sensor sample are provided in Supplementary Figure S1.

### Material testing and environmental conditioning

The mechanical testing system comprises a material testing system (Instron 3345) fitted with a custom-built environmental chamber (Model 5500-8485; ETS). An image and schematic of the setup are provided in Figure 1b and c, respectively. The environmental chamber has a temperature range of −40°C to 80°C and a humidity range of <5% to >95% at room temperature. For the temperature tests, samples were left in the environmental chamber for at least 10 min before testing, as prescribed by ASTM D412. For any humidity tests, samples were left in the environmental chamber overnight (16–24 h).

Three different characterization tests were performed: pull-to-failure tests, stiffness tests, and relaxation tests. The temperature dependence was determined between −40°C and 80°C at increments of 20°C, except for the relaxation tests, which were only conducted at −40°C, 20°C, and 80°C. The humidity dependence on material properties was determined by conducting tests at 95% RH, 40–60% RH (room conditions), and 5% RH.

An Instron 2530—1 kN load cell was used for the pull-to-failure and stiffness tests. An Instron 2519—50 N load cell was used for the relaxation tests because of its much lower creep specification (±0.1% of force capacity over a period of 20 min, compared with ±0.1% of force capacity over a period of 3 min for the 2530—1 kN). Both load cells have a linearity and repeatability of ±0.25% of the reading (from 0.2/0.5 to 100% of force capacity for the 2530—1 kN/2519—50 N, respectively).

For the stiffness tests, EcoFlex and Dragon Skin samples were strained to ∼300% and the Smooth-Sil and Sylgard samples to ∼100% at a rate of 500 mm/min. Sylgard samples under certain conditions (i.e., change in humidity, higher temperature conditions) exhibited failure before the preset 100% strain. In this case, the maximum strain was reduced to 80% to prevent failure. Before a stiffness test, samples were subjected to a preload to remove slack in the sample and a prestretch cycle at 1000 mm/min to remove the Mullin's effect.^27^ A faster rate was chosen here than for the recorded tests to increase the testing efficiency. The preloads were 0.04, 0.1, 0.4, and 0.4 N for EcoFlex, Dragon Skin, Smooth-Sil, and Sylgard, respectively, and the maximum strains during the prestretch cycle were ∼350% for the EcoFlex and Dragon Skin samples, and 120% for the Smooth-Sil and Sylgard samples.

For the failure tests, samples were stretched to failure at a rate of 500 mm/min. A small preload of 0.02, 0.1, 0.2, and 0.2 N was applied to the EcoFlex, Dragon Skin, Smooth-Sil, and Sylgard samples, respectively, to remove slack in the samples before testing.

In the stress-relaxation tests, EcoFlex and Dragon Skin samples were stretched to ∼90–100% strain and Smooth-Sil and Sylgard to ∼40–50% strain at a strain rate of 1000 mm/min, and held in the stretched position for 30 min. The stress-relaxation procedure was based on earlier work^18^ and follows the guidance of ASTM E328-21, which describes a general procedure for stress-relaxation tests. The rate was set to the maximum rate of the testing system (1000 mm/min) to increase testing efficiency and to create a high viscoelastic response. The relaxation percentage was determined by comparing the stress at the beginning of the holding period with the final stress at the end of the test.

The beginning of the holding period was defined as the point at which the final displacement was first reached to eliminate stress fluctuations due to the displacement control of the testing system. The final stress was calculated as the average over the final ∼3.5 min of the test to account for thermal fluctuations caused by the heating/cooling control of the environmental chamber.

Images of the samples were captured with an imaging system (Grasshopper 3; Point Grey Research) every second to analyze the real strain in the gauge area using the painted dots. The relationship between the measured displacement from the Instron and the measured strain from the images was obtained through a cubic fit of the data, with typical *R*^2^ values >0.99. A cubic fit was chosen because this was the lowest order fit that yielded a fit through the origin. The strain measurements showed a deviation of ±2%, which includes any variations caused by sample loading. Most images taken at subzero temperatures were not usable due to the liquid nitrogen mist in the chamber, except for the failure tests at 0°C for Dragon Skin, Smooth-Sil, and Sylgard. An average fit of all the data above 0°C was used when the images were not usable.

The strains at which most EcoFlex and Dragon Skin samples broke were too large to be captured within the field of view of the camera. Six additional samples per material were tested with a sufficiently large field of view. This setup was, however, not feasible to perform all tests because of a much more labor-intensive image postprocessing and restrictions on laboratory space. The resulting average fit of these large field of view tests was used to fit all EcoFlex and Dragon Skin data.

### Sensor tests

Dragon Skin sensors were stretched to ∼100% strain and Smooth-Sil sensors to ∼50% strain at a rate of 5 mm/s using the material testing system. The average strain was calculated from the initial length of the sensor at the start of the test. The capacitance of the sensors was recorded with an LCR meter (E4980AL; Keysight Technologies) at an excitation frequency of 200 Hz. Sensors were acclimatized in the same way as the elastomer samples.

## Results and Discussion

### Stiffness response to environmental conditions

**FIG. 2.**
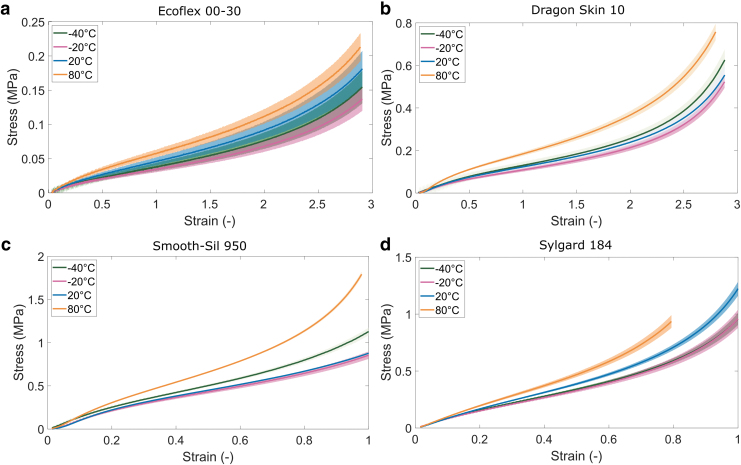
Stress–strain curves at different temperature conditions for **(a)** EcoFlex 00-30; **(b)** Dragon Skin 10; **(c)** Smooth-Sil 950; and **(d)** Sylgard 184. Shaded areas represent standard deviations (*n* = 6).

Figure 2 shows the stress–strain curves of all materials under different thermal conditions. For clarity purposes, only the data at −40°C, −20°C, 20°C, and 80°C are shown. The data at these temperatures are representative of the observed trends. The additional data at temperatures 0°C, 40°C, and 60°C are available in Supplementary Figure S2. Sylgard was only tested to 80% strain at 80°C due to early failure of the material. The graphs show that, in general, the higher the temperature the stiffer the material. Although the EcoFlex 00-30 measurements show relatively large variation, the difference between low-temperature (−20°C) and high-temperature (80°C) measurements is sufficiently large to conclude there is a temperature effect.

An increasing stiffness with temperature is reported for various unfilled elastomers^23,28,29^ and can be attributed to entropic-related energy.^28,30,31^ The configuration entropy of elastomers increases with temperature, enabling the molecules to return to a less ordered state,^31^ leading to an overall contraction of the material and higher stresses.^28,30,31^ This stiffening effect can potentially be offset or reversed by the presence of reinforcing fillers in elastomers, reducing the contribution of the entropic-related elastomer response.^32^ We further note that temperature effects may present differently in the first stretch cycle compared with the subsequent stretch cycles,^23^ which is not captured in our data because we used a prestretch cycle to remove the Mullins effect from the measurements.

The exception to the general trend of increasing stiffness with increasing temperature is the measurements at −40°C for Smooth-Sil, Dragon Skin, and EcoFlex. These materials exhibit higher stiffness at −40°C than at −20°C (Fig. 2a–c). The distinction between the EcoFlex measurements is less clear because of the relatively large variation in the EcoFlex tests, resulting in overlapping confidence intervals between the −40°C and −20°C tests.

A potential explanation for an increase in stiffness with decreasing temperature is a transition to a glassy state of the elastomer.^29^ Although the glass transition temperature is not reported in the technical data supplied from the manufacturer, EcoFlex is known to have a glass transition temperature around −30°C.^33,34^ The technical data from the manufacturer list the same useful temperature range for Dragon Skin and Smooth-Sil as for EcoFlex (−65°F to 450°F, −54°C to 232°C), and so, it is likely their glass transition temperatures are similar too. Changes in the mechanical properties due to glass transition can gradually take place over a temperature range of 50°C,^30^ which may explain the relatively small changes in stiffness. No stiffening effect was observed for the Sylgard 184, which has a much lower glass transition temperature around −120°C.^35,36^

**FIG. 3.**
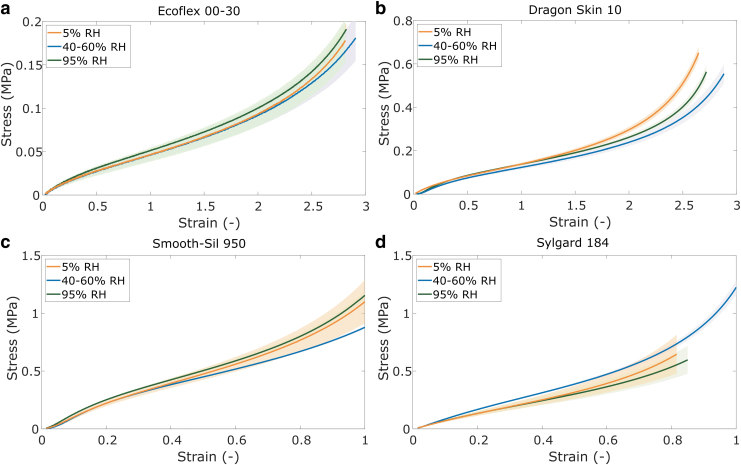
Stress–strain curves at different humidity conditions for **(a)** EcoFlex 00-30; **(b)** Dragon Skin 10; **(c)** Smooth-Sil 950; and **(d)** Sylgard 184. Shaded areas represent standard deviations (*n* = 6).

The effect of humidity on respective material properties is exhibited in Figure 3. No clear trend is observed between the stiffness of the materials and the humidity conditions. While Dragon Skin and Smooth-Sil appear to show marginally higher stiffnesses when samples are subject to either low (5%) or high humidity (95%), the differences are not as substantial as those found in temperature changes. The differences in stiffness in EcoFlex, Smooth-Sil, and Sylgard are too small compared with the relatively large standard deviations to attribute to changes in humidity. Both Dragon Skin and Smooth-Sil show the lowest stiffness at ambient room humidity. A decreasing stiffness with increasing humidity was observed for a VHB elastomer,^37^ which means the humidity response likely depends on the type of elastomer.

### Failure response to environmental conditions

**FIG. 4.**
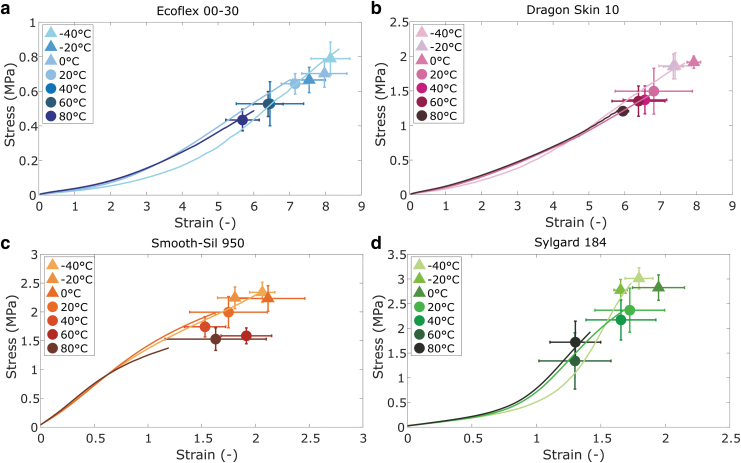
Failure tests at different temperature conditions for **(a)** EcoFlex 00-30; **(b)** Dragon Skin 10; **(c)** Smooth-Sil 950; and **(d)** Sylgard 184. Representative example curves are shown for −40°C, 0°C, and 80°C to guide the reader. The icons represent average stress and strain at failure, and the error bars represent standard deviations (*n* = 6).

Figure 4 shows the effect of temperature on the failure tests. In general, as the temperature increases, the failure stress and strain of the materials decrease. Although the differences between consecutive temperature intervals are not always distinguishable due to the overlapping standard deviations, the general trend of higher failure stress and strain at lower temperatures is clearly visible in the graphs. Comparable trends with both a reduced energy input and strain at break with increasing temperature are reported for other elastomers in a similar temperature range.^38,39^ Early failure, similar to our observation for Sylgard in the stress–strain tests, was observed in tests at elevated temperatures.^23,40^

**FIG. 5.**
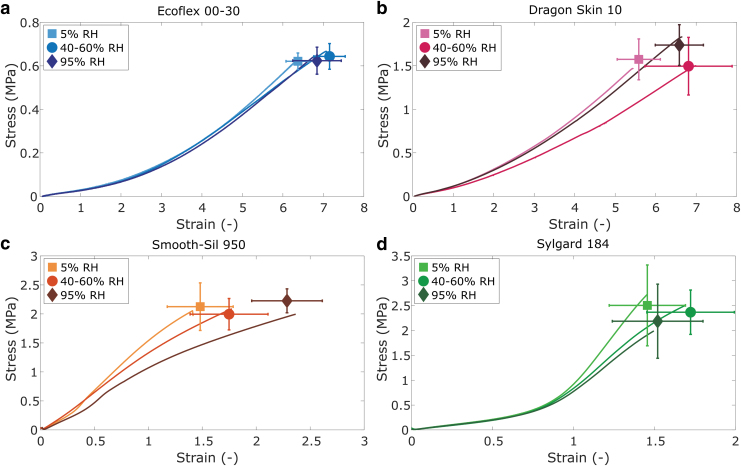
Failure tests at different humidity conditions for **(a)** EcoFlex 00-30; **(b)** Dragon Skin 10; **(c)** Smooth-Sil 950; and **(d)** Sylgard 184. Representative example curves are shown to guide the reader. The icons represent average stress and strain at failure and the error bars represent standard deviations (*n* = 6).

The effect of humidity on failure tests is shown in Figure 5. The differences in failure stress and strain are very small or negligible for all elastomers. The early failure observed in some of the Sylgard stiffness tests (Fig. 3d) is therefore likely unrelated to the humidity conditions and due to variation in the samples. The most noticeable difference is observed for the Smooth-Sil samples at 95% RH compared with room conditions (Fig. 5c). Although the stress at failure is very similar, the strain at break is slightly higher. A lower modulus may be expected for swollen elastomers,^30^ but this is not what we observed in the stiffness tests for Smooth-Sil.

Previous work^18^ showed that batch-to-batch differences in the strain at break for Smooth-Sil are relatively large with a 75% strain difference between batches (300–375% strain). Although we attempted to minimize batch effects by selecting samples from at least two different batches in one test group, these batches may be different between test groups. Due to the relatively large standard deviation in these tests and the lack of a similar trend in the other elastomers, the differences may be due to large differences between batches.

### Relaxation response to environmental conditions

**FIG. 6.**
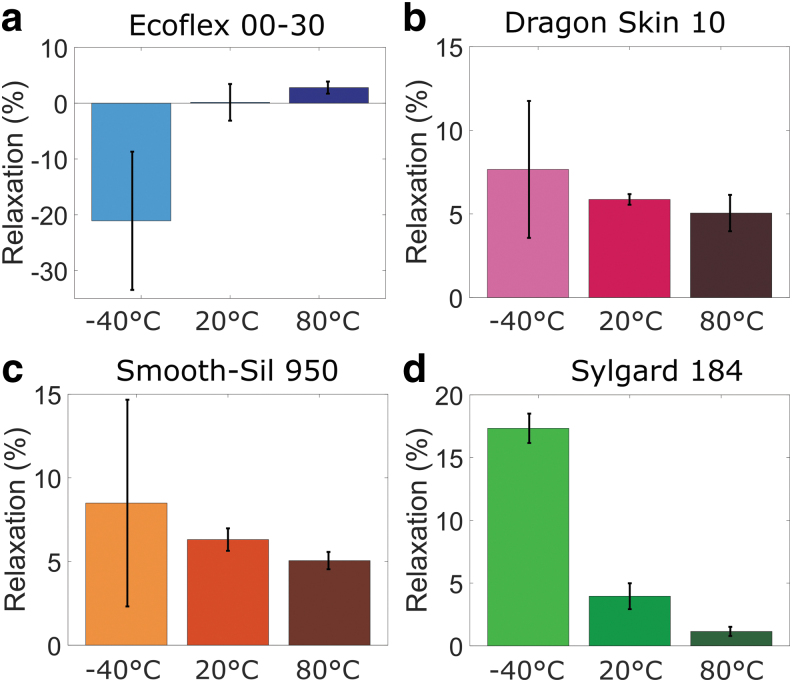
Relaxation tests at different temperature conditions for **(a)** EcoFlex 00-30; **(b)** Dragon Skin 10; **(c)** Smooth-Sil 950; and **(d)** Sylgard 184. Error bars represent standard deviations (*n* = 6).

**FIG. 7.**
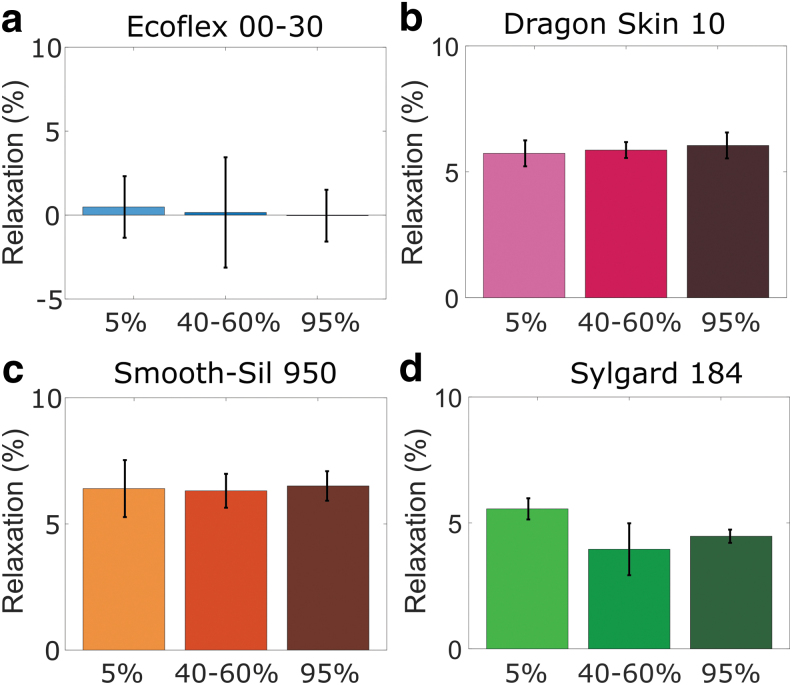
Relaxation tests at different humidity conditions for **(a)** EcoFlex 00-30; **(b)** Dragon Skin 10; **(c)** Smooth-Sil 950; and **(d)** Sylgard 184. Error bars represent standard deviations (*n* = 6).

Figures 6 and 7 show the average relaxation percentages after 30 min of relaxation for different temperature and humidity conditions, respectively. The graphs indicate that the relaxation behavior is dependent on temperature, but not on humidity. A small increase in relaxation at low humidity is observed for Sylgard (Fig. 7d), but this small change likely does not require special consideration in the design process of a soft robot. The relaxation behavior of all four materials is most sensitive to freezing temperatures (−40°C) compared with elevated temperatures (80°C).

The most notable difference in relaxation behavior based on temperature is observed between the freezing and room temperature conditions (Fig. 6). Sylgard has an increased average relaxation that is about five times as high as the relaxation observed at room temperature (Fig. 6d), whereas EcoFlex shows stiffening rather than relaxing behavior (Fig. 6a). Stress recovery after stress softening in the initial strain cycles (Mullins effect) is also a temperature- and time-dependent process with recovery times decreasing with increasing temperature.^41^ The change in relaxation between 20°C and 80°C is relatively small for all elastomers. The standard deviation of the tests performed at −40°C for Dragon Skin and Smooth-Sil is too big to attribute the larger average relaxation to the temperature effect with certainty (Fig. 6b, c).

Relaxation behavior is highly dependent on the type of polymer,^42^ but the observed trend of increasing relaxation with decreasing temperature in the current work is in line with observations on several other elastomers.^29,40^

The stiffening behavior that is observed for EcoFlex may be explained by the glass transition temperature around −30°C.^33,34^ Extreme stiffening behavior is observed for one of the EcoFlex samples at the lowest temperatures of the cooling cycle (−45°C) with the maximum stress during relaxation as high as four times the initial stress. Similar extreme stiffening behavior is observed for two Dragon Skin samples (Supplementary Fig. S3). These samples were all frozen solid when removed from the test setup, and are removed from the presented data as outliers. Sylgard has a much lower glass transition temperature (around −120°C^35,36^), and no stiffening effect was observed.

### Effect of extreme temperature exposure

Since lunar conditions can exhibit fluctuations in temperature ranging from −230°C to 120°C, and the environmental chamber is not equipped to reach these extreme temperatures, additional tests were performed to investigate the effect of extreme temperature exposure. The aim is to identify potential long-term changes in material properties that can affect the usability of the materials in space.

**FIG. 8.**
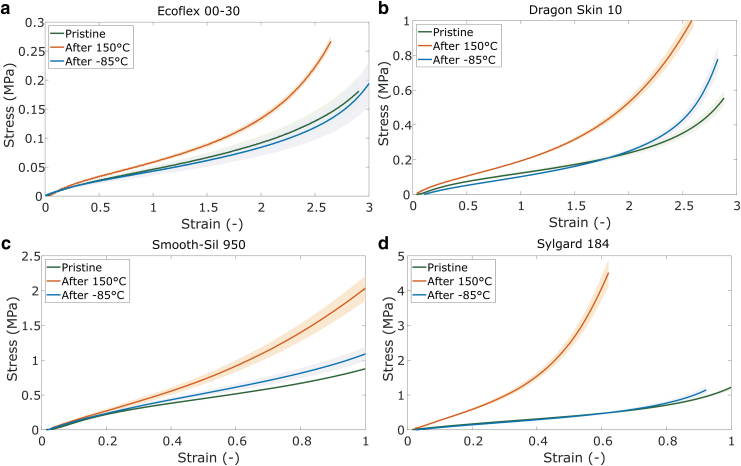
Stress–strain curves of samples before and after thermal treatment at 150° C and −85°C for **(a)** EcoFlex 00-30; **(b)** Dragon Skin 10; **(c)** Smooth-Sil 950; and **(d)** Sylgard 184. Shaded areas represent standard deviations (*n* = 6).

**FIG. 9.**
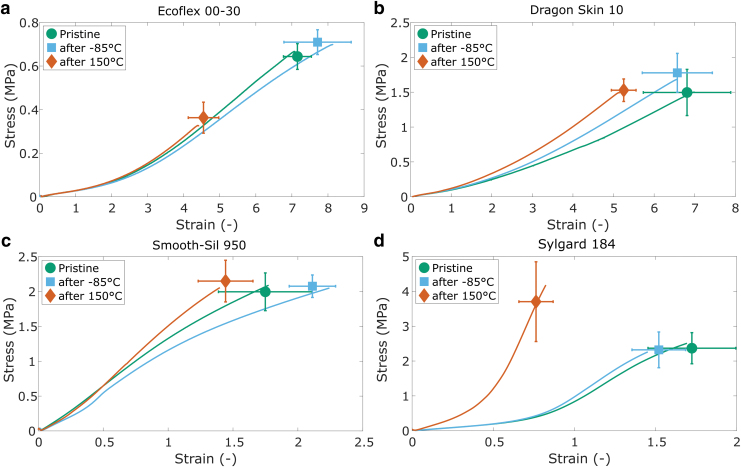
Failure tests after thermal treatment at 150°C and −85°C compared with tests without any treatment for **(a)** EcoFlex 00-30; **(b)** Dragon Skin 10; **(c)** Smooth-Sil 950; and **(d)** Sylgard 184. Representative example curves are shown to guide the reader. The icons represent average stress and strain at failure, and the error bars represent standard deviations (*n* = 6).

Two additional sets of samples were tested per material; one set was heated at 150°C and the other set was cooled at −85°C overnight (16 h). All tests were performed at room temperature, at least 30 min after the thermal treatment. For the stiffness tests, all samples were tested before and after thermal treatment. The effect of extreme temperature exposure on the stress–strain and failure behavior is displayed in Figures 8 and 9, respectively. The “pristine” data are repeated from the stress–strain and failure studies (Figs. 2 and 4). Separate data of the same samples before and after thermal treatment are available in Supplementary Figures S4 and S5.

The most substantial changes to mechanical properties are observed when samples are subject to heat treatment, where all materials undergo a permanent increase in stiffness (Fig. 8). An increase in stiffness after heat treatment agrees with previous works,^21^ as the chemical crosslinking of PDMS can be aided by an increase in temperature. All the materials tested are silicones that undergo an additional reaction between vinyl groups and silane functionalized PDMS, catalyzed by organometallic platinum. Heating up this system accelerates the ability for platinum catalysts to react with the respective vinyl and PDMS groups, enabling a higher degree of crosslinking and thus higher stiffness. In addition, Sylgard and Ecoflex samples fail at much lower strains than pristine samples (Fig. 9).

The effect of a freeze–thaw cycle on all materials is not as large compared with the heat treatment, with only small changes observed in the stiffness of Smooth Sil and Dragon Skin. A direct comparison of the pre- and postfreezing stiffness curves for Dragon Skin (Supplementary Fig. S5b) shows no effect of the freeze–thaw procedure and the small deviation may have resulted from batch or test differences. The elevated stiffness after freeze–thaw cycles may relate to cold crystallization,^43,44^ where molecular crystallization leads to an increase in crosslinking density. Silicones have been shown to exhibit a cold crystallization phase around approximately −70°C,^43,44^ which may explain the increased stiffness of the Smooth-Sil samples after freezing.

Although these tests do not describe the material behaviors at the more extreme temperatures (as low as −85°C and up to 150°C), they show that material integrity is preserved after exposure and stiffness changes can be expected, which is the first step in gaining a full understanding of the material characteristics in lunar conditions.

### Sensor case study

Capacitive sensors can be used to measure strain since their measured capacitance directly relates to the geometry of the sensor:

(1) $$C=\varepsilon_{0}\varepsilon_{r}\frac{Lw}{t}$$

where $\varepsilon_{0}$ and $\varepsilon_{r}$ are the free space and relative permittivity, respectively; *L* and *w* are the length and width of the electrode area, and *t* is the thickness of the dielectric layer. Since the measured capacitance of elastomer-based sensors can change based on the environmental conditions,^15,45^ capacitive sensors were chosen for a case study on the performance of a soft robotic component under different environmental conditions. The sensors were made with Dragon Skin or Smooth-Sil as base material. The Dragon Skin sensors were used for a temperature study and the Smooth-Sil sensors for a humidity study.

**FIG. 10.**
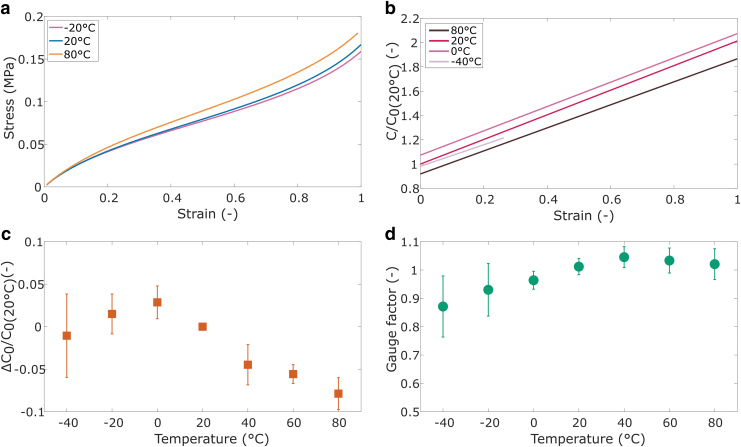
Sensor tests at different temperature conditions: **(a)** average stiffness; **(b)** average capacitance normalized against the initial capacitance at 20°C (C_0(20°C)_); **(c)** relative difference between C_0_ and C_0(20°C)_; and **(d)** gauge factor. Error bars represent standard deviations (*n* = 6 for all tests, except at −40°C [*n* = 4]).

The results of the temperature study are captured in Figure 10. The sensors showed increased stiffness with increasing temperature between −20°C and 80°C (Fig. 10a), which was expected based on the elastomer tests. The average stiffness increase between −20°C and 80°C was, however, not as large (about 15%) as for the neat elastomer (about 70%). The graphite in the graphite-filled electrodes could explain the reduced temperature response, since the stiffness of the graphite may decrease with temperature in the tested temperature range.^46–48^

Figure 10b shows the linear relationship between capacitance and strain at four representative temperatures (all data are included in Supplementary Fig. S6), and Figure 10c shows the relative capacitance change at 0% strain for all temperatures. Both graphs show a trend of increasing capacitance with decreasing temperature between 0°C and 80°C and decreasing capacitance with decreasing temperature between −40°C and 0°C. We observed a reduced linear strain region at temperatures below 0°C, which is represented by the shorter capacitance–strain curve at −40°C (Fig. 10a).

Assuming the geometry of all sensors is the same at 0% strain, the change in capacitance is most likely caused by a change in the dielectric constant ($C_{0}\propto \varepsilon_{r}$). The dielectric constant of elastomers is known to be temperature dependent and similar trends are reported for other elastomers^49–51^ and capacitive sensors.^15^ The dielectric constant can be related to dipole polarization, which becomes easier with increasing temperature and thus increases the dielectric constant.^49,50^ The observed peak in the initial capacitance relates to the temperature at which increased thermal motion of the molecules reduces the polarization, leading to a subsequent decrease in dielectric constant.^49^

The sensor sensitivity, represented by the gauge factor (GF = (C/ΔC)/ξ), does not show a clear trend based on temperature (Fig. 10d) with values very close to 1. The average GFs at −20°C and −40°C are slightly lower, but have a large standard deviation. Additional tests were performed to measure the Poisson's ratio of both the sensors and Dragon Skin at 0°C and 20°C, since the sensitivity of the sensors is determined by the Poisson's ratio of the dielectric layer.^14^ No evidence was found for a relationship between the Poisson's ratio and temperature that can explain the change in GF, and so, the small deviations can likely be attributed to normal deviations between tests.

The overall sensor performance reduces at temperatures below 0°C. At temperatures of 20°C and above, all sensors show a linear capacitance response with strain to at least 100%. Below 0°C, the sensors show reduced linearity with strain. At 0°C, one of the sensors shows reduced linearity after about 80% strain. At −40°C, two sensors did not produce any reliable sensor measurements, and the other sensors had a maximum measurement range between 20% and 70% strain.

We observe an increasing electrode resistance of the sensors with decreasing temperature (Supplementary Fig. S7), which is in line with observations on other conductive polymers, although at temperatures above 0°C.^15^ A high electrode resistance can affect the maximum measurement range of elastomer capacitance sensors,^52,53^ and the increasing resistance with temperature explains the reduced performance of the sensors. The breakdown voltage of dielectric elastomers is also temperature dependent,^54^ but this is unlikely to affect the sensor performance since these breakdown voltages are of a different order of magnitude (kV) compared with the voltages that are relevant to capacitive sensors (several V).

**FIG. 11.**
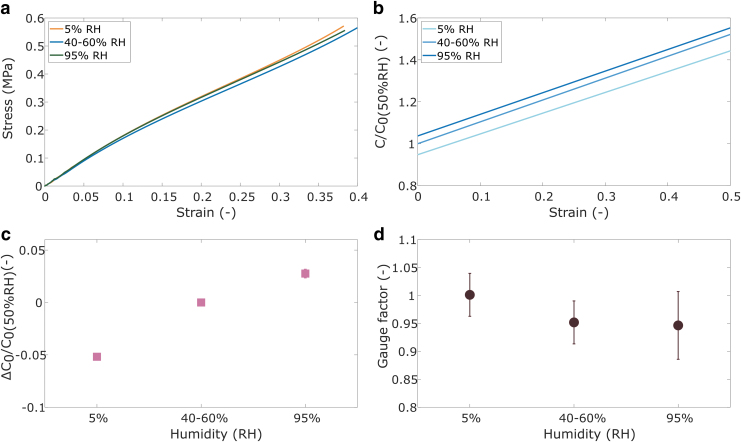
Sensor tests at different humidity conditions: **(a)** average stiffness; **(b)** average capacitance normalized against the initial capacitance at 40–60% RH (C_0(50%RH)_); **(c)** relative difference between C_0_ and C_0(50%RH)_; and **(d)** gauge factor. Error bars represent standard deviations (*n* = 7 for all tests).

The results of the humidity study are shown in Figure 11. No change in the sensor stiffness was observed based on humidity (Fig. 11a), which is in line with observations from the elastomer tests. The measured capacitance normalized against the initial capacitance at room conditions shows small changes with humidity (Fig. 11b). The most observable difference is the change of the initial capacitance with humidity (Fig. 11c). The lowest humidity results in the lowest initial capacitance and the highest humidity results in the highest initial capacitance. The GF for all humidities are all very close to 1 (Fig. 11d). The observation of increasing capacitance with increasing humidity agrees with other studies that show increased capacitance when capacitive sensors are in contact with water,^45,55^ due to increased current leakage or space charge.^45,56^

The sensor case study shows that the constituent material characterization can be used in the design of soft robotic components, but that the composition and function of the component need to be carefully considered. The characterization in this study focuses on the mechanical properties of the elastomers. The sensors performed as expected with increasing stiffness with increasing temperature and with limited effects based on humidity. However, the composite nature of the sensors likely affected the temperature response, which means further characterization of all constituent materials is required to accurately predict the behavior of composite structures. The properties specific to sensor design, such as the electrode resistance and dielectric constant, were not captured in the mechanical study. This means that component-specific properties may need separate characterization to predict their full performance.

## Conclusion

This work investigated the mechanical behavior of four commonly used elastomers in soft robotics over a range of temperature and humidity conditions. A case study on elastomer capacitive sensors was performed to showcase the performance of a soft robotic component in various conditions. In general, this study showed that the operating conditions should be considered in the design and testing of elastomers in soft robot applications.

Three main conclusions can be drawn. First, we observed temperature-dependent behavior for all tested elastomers, which presented as increasing stiffness and decreasing strain at failure with increasing temperatures. No distinct effects were observed for changes in humidity. Second, extreme stiffening behavior was observed at −40°C during prolonged exposure in several stress-relaxation tests. Although the materials seem to be able to withstand the low temperatures, active use below the glass transition temperature should be avoided to prevent early failure.

Third, the elastomer-based sensor performance was dependent on both humidity and temperature conditions. The sensors showed increasing stiffness with temperature, but this increase did not precisely match that of the constituent elastomers, indicating that composite materials should be independently characterized for high-fidelity performance predictions. Furthermore, the sensors showed both a temperature and humidity dependence due to electric and dielectric impacts, while the constituent elastomers were relatively humidity-independent, which speaks to the importance of considering mechanical and electrical effects in tandem for electromechanical components. These insights will aid the design for a successful soft robotic operation outside a laboratory setting.

## Supplementary Material

Supplemental data

Supplemental data

Supplemental data

Supplemental data

Supplemental data

Supplemental data

Supplemental data

## References

[1] Shah DS, Powers JP, Tilton LG, et al. A soft robot that adapts to environments through shape change. Nat Mach Intell 2021;3(1):51–59; doi: 10.1038/s42256-020-00263-1

[2] Kriegman S, Walker S, Shah D, et al. Automated shapeshifting for function recovery in damaged robots. In: Proceedings of Robotics: Science and Systems, Freiburg im Breisgau, Germany, 2019; doi: 10.15607/rss.2019.xv.028

[3] Umedachi T, Vikas V, Trimmer BA. Softworms: The design and control of non-pneumatic, 3D-printed, deformable robots. Bioinspir Biomim 2016;11(2):025001; doi: 10.1088/1748-3190/11/2/025001 26963596

[4] Yang X, Shang W, Lu H, et al. An agglutinate magnetic spray transforms inanimate objects into millirobots for biomedical applications. Sci Robot 2020;5(48):1–13; doi: 10.1126/scirobotics.abc8191 33208522

[5] Zhang Y, Li P, Quan J, et al. Progress, challenges, and prospects of soft robotics for space applications. Adv Intell Syst 2022;5(3):2200071; doi: 10.1002/aisy.202200071

[6] Buckner TL, Bilodeau RA, Yup S, et al. Roboticizing fabric by integrating functional fibers. PNAS 2020;117(41):25360–25369; doi: 10.1073/pnas.2006211117 32989123 PMC7568323

[7] European Space Agency. Moon; 2019. Available from: https://sci.esa.int/s/ABkdpow [Last accessed: December 24, 2021].

[8] NASA. Earth's Moon—Inside & Out; n.d. Available from: https://moon.nasa.gov/inside-and-out/overview/ [Last accessed: December 24, 2021].

[9] Dai A. Recent climatology, variability, and trends in global surface humidity. J Clim 2006;19(15):3589–3606; doi: 10.1175/JCLI3816.1

[10] Miriyev A, Stack K, Lipson H. Soft material for soft actuators. Nat Commun 2017;8(1):1–8; doi: 10.1038/s41467-017-00685-3 28928384 PMC5605691

[11] Bilodeau RA, Miriyev A, Lipson H, et al. All-Soft Material System for Strong Soft Actuators. In: IEEE International Conference on Soft Robotics, Livorno, Italy; 2018; pp. 288–294; doi: 10.1109/ROBOSOFT.2018.8404934

[12] Duduta M, Clarke DR, Wood RJ. A High Speed Soft Robot Based on Dielectric Elastomer Actuators. In: Proceedings of IEEE International Conference on Robotics and Automation, Singapore; 2017; pp. 4346–4351; doi: 10.1109/ICRA.2017.7989501

[13] Li G, Chen X, Zhou F, et al. Self-powered soft robot in the Mariana Trench. Nature 2021;591(7848):66–71; doi: 10.1038/s41586-020-03153-z 33658693

[14] Porte E, Kramer-Bottiglio R. Nonlinear Poisson's ratio for modeling hyperelastic capacitive sensors. Adv Mater Technol 2021;6(8):2001247; doi: 10.1002/admt.202001247

[15] Shintake J, Piskarev E, Jeong SH, et al. Ultrastretchable strain sensors using carbon black-filled elastomer composites and comparison of capacitive versus resistive sensors. Adv Mater Technol 2018;3(3):1700284; doi: 10.1002/admt.201700284

[16] Atalay O, Atalay A, Gafford J, et al. A highly stretchable capacitive-based strain sensor based on metal deposition and laser rastering. Adv Mater Technol 2017;2(9):1700081; doi: 10.1002/admt.201700081

[17] White EL, Yuen MC, Case JC, et al. Low-cost, facile, and scalable manufacturing of capacitive sensors for soft systems. Adv Mater Technol 2017;2(9):1700072; doi: 10.1002/admt.201700072

[18] Case JC, White EL, Kramer RK. Soft material characterization for robotic applications. Soft Robot 2015;2(2):80–87; doi: 10.1089/soro.2015.0002

[19] Marechal L, Balland P, Lindenroth L, et al. Toward a common framework and database of materials for soft robotics. Soft Robot 2021;8(3):284–297; doi: 10.1089/soro.2019.0115 32589507

[20] Park S, Mondal K, Treadway RM, et al. Silicones for stretchable and durable soft devices: Beyond Sylgard-184. ACS Appl Mater Interfaces 2018;10(13):11261–11268; doi: 10.1021/acsami.7b18394 29578686

[21] Johnston ID, McCluskey DK, Tan CKL, et al. Mechanical characterization of bulk Sylgard 184 for microfluidics and microengineering. J Micromech Microeng 2014;24(3):035017; doi: 10.1088/0960-1317/24/3/035017

[22] Santiago-Alvarado A, Cruz-Félix AS, González-García J, et al. Polynomial fitting techniques applied to opto-mechanical properties of PDMS Sylgard 184 for given curing parameters. Mater Res Express 2020;7(4):045301; doi: 10.1088/2053-1591/ab8339

[23] Liao Z, Hossain M, Yao X, et al. A comprehensive thermo-viscoelastic experimental investigation of Ecoflex polymer. Polym Test 2020;86:106478; doi: 10.1016/j.polymertesting.2020.106478

[24] Nielsen TB, Hansen CM. Elastomer swelling and Hansen solubility parameters. Polym Test 2005;24(8):1054–1061; doi: 10.1016/j.polymertesting.2005.05.007

[25] Zhang Y, He P, Zou Q, et al. Preparation and properties of water-swellable elastomer. J Appl Polym Sci 2004;93(4):1719–1723; doi: 10.1002/app.20633

[26] Akhtar M, Qamar SZ, Pervez T, et al. Performance evaluation of swelling elastomer seals. J Pet Sci Eng 2018;165:127–135; doi: 10.1016/j.petrol.2018.01.064

[27] Diani J, Fayolle B, Gilormini P. A review on the Mullins effect. Eur Polym J 2009;45(3):601–612; doi: 10.1016/j.eurpolymj.2008.11.017

[28] Li X, Bai T, Li Z, et al. Influence of the temperature on the hyper-elastic mechanical behavior of carbon black filled natural rubbers. Mech Mater 2016;95:136–145; doi: 10.1016/j.mechmat.2016.01.010

[29] Rey T, Chagnon G, Le Cam JB, et al. Influence of the temperature on the mechanical behaviour of filled and unfilled silicone rubbers. Polym Test 2013;32(3):492–501; doi: 10.1016/j.polymertesting.2013.01.008

[30] Treloar LRG. Physics of Rubber Elasticity. 3rd ed. Oxford University Press: Oxford; 2005.

[31] Anthony RL, Caston RH, Guth E. Equations of state for natural and synthetic rubber-like materials. J Phys Chem 1942;46(8):826–840.

[32] Lavazza J, Contino M, Marano C. Strain rate, temperature and deformation state effect on Ecoflex 00-50 silicone mechanical behaviour. Mech Mater 2023;178:104560; doi: 10.1016/j.mechmat.2023.104560

[33] Spiridon I, Anghel NC, Darie-Nita RN, et al. New composites based on starch/Ecoflex^®^/biomass wastes: Mechanical, thermal, morphological and antimicrobial properties. Int J Biol Macromol 2020;156:1435–1444; doi: 10.1016/j.ijbiomac.2019.11.185 31770560

[34] Fang Y, Li Y, Wang X, et al. Cryo-transferred ultrathin and stretchable epidermal electrodes. Small 2020;16(28):1–8; doi: 10.1002/smll.202000450 32529803

[35] Brounstein Z, Zhao J, Geller D, et al. Long-term thermal aging of modified sylgard 184 formulations. Polymers (Basel) 2021;13(18):3125; doi: 10.3390/polym13183125 34578026 PMC8466950

[36] Lahiff E, Leahy R, Coleman JN, et al. Physical properties of novel free-standing polymer-nanotube thin films. Carbon N Y 2006;44(8):1525–1529; doi: 10.1016/j.carbon.2005.12.018

[37] Zhang J, Liu X, Liu L, et al. Modeling and experimental study on dielectric elastomers incorporating humidity effect. EPL 2020;129(5):57002; doi: 10.1209/0295-5075/129/57002

[38] Smith TL. Ultimate tensile properties of elastomers. I. Characterization by a time and temperature independent failure envelope. Rubber Chem Technol 1964;37(4):777–791; doi: 10.5254/1.3540377

[39] Whittaker RE. Tensile failure properties of some branched polyurethane elastomers. Polymer (Guildf) 1972;13(4):169–173; doi: 10.1016/0032-3861(72)90041-9

[40] Ilseng A, Skallerud BH, Clausen AH. Tension behaviour of HNBR and FKM elastomers for a wide range of temperatures. Polym Test 2016;49:128–136; doi: 10.1016/j.polymertesting.2015.11.017

[41] Liao Z, Yang J, Hossain M, et al. The time and temperature dependences of the stress recovery of Ecoflex polymer. Int J Non Linear Mech 2023;149:104338; doi: 10.1016/j.ijnonlinmec.2022.104338

[42] Tobolsky A V. Stress relaxation studies of the viscoelastic properties of polymers. J Appl Phys 1956;27(7):673–685; doi: 10.1063/1.1722465

[43] Stricher AM, Rinaldi RG, Barrès C, et al. How I met your elastomers: From network topology to mechanical behaviours of conventional silicone materials. RSC Adv 2015;5(66):53713–53725; doi: 10.1039/c5ra06965c

[44] Weir CE, Leser WH, Wood LA. Crystallization and second-order transitions in silicone rubbers. J Res Natl Bur Stand (1934) 1950;44:367–372; doi: 10.5254/1.3543067

[45] Walker C, Anderson I. From land to water: Bringing dielectric elastomer sensing to the underwater realm. Electroact Polym Actuat Dev 2016;9798:97982B; doi: 10.1117/12.2218975

[46] Mason IB, Knibbs RH. Variation with temperature of Young's modulus of polycristalline graphite. Nature 1960;188(4744):33–35; doi: 10.1038/188033a0

[47] Marrow TJ, Šulak I, Li BS, et al. High temperature spherical nano-indentation of graphite crystals. Carbon N Y 2022;191:236–242; doi: 10.1016/j.carbon.2022.01.067

[48] Mason IB, Knibbs RH. The Young's modulus of carbon and graphite artefacts. Carbon N Y 1967;5:493–506; doi: 10.1016/0008-6223(67)90026-7

[49] Sheng J, Chen H, Li B, et al. Temperature dependence of the dielectric constant of acrylic dielectric elastomer. Appl Phys A Mater Sci Process 2013;110(2):511–515; doi: 10.1007/s00339-012-7254-2

[50] Jean-Mistral C, Sylvestre A, Basrour S, et al. Dielectric properties of polyacrylate thick films used in sensors and actuators. Smart Mater Struct 2010;19(7):075019; doi: 10.1088/0964-1726/19/7/075019

[51] Zhang QM, Su J, Kim CH, et al. An experimental investigation of electromechanical response in a polyurethane elastomer. J Appl Phys 1997;81(6):2770–2776; doi: 10.1063/1.363981

[52] Porte E, Sipple T, Sanchez Botero L, et al. Capacitive Sensor Measurement Rate Improves by Pre-Stretching. In: IEEE International Conference on Soft Robotics, New Haven, CT, USA; 2021; pp. 412–418; doi: 10.1109/RoboSoft51838.2021.9479328

[53] Tairych A, Anderson IA. Capacitive stretch sensing for robotic skins. Soft Robot 2019;6(3):389–398; doi: 10.1089/soro.2018.0055 31074690

[54] Zhang J, Sheng J, Liu X, et al. Temperature effect on electromechanical properties of polyacrylic dielectric elastomer: An experimental study. Smart Mater Struct 2020;29(4):047002; doi: 10.1088/1361-665X/ab79b7

[55] Shrestha M, Lu Z, Lau G. Sensors and Actuators: B. Chemical High humidity sensing by ‘hygromorphic’ dielectric elastomer actuator. Sens Actuators B Chem 2021;329:129268; doi: 10.1016/j.snb.2020.129268

[56] Madsen FB, Daugaard AE, Hvilsted S, et al. The current state of silicone-based dielectric elastomer transducers. Macromol Rapid Commun 2016;37(5):378–413; doi: 10.1002/marc.201500576 26773231

