# Supplementary figures and images for: Characterization of Temperature and Humidity Dependence in Soft Elastomer Behavior
Source: Soft Robot. 2024 Feb 13;11(1):118–30. doi: 10.1089/soro.2023.0004 (PMC10880277; doi:10.1089/soro.2023.0004)

# Schematic of layered sensor


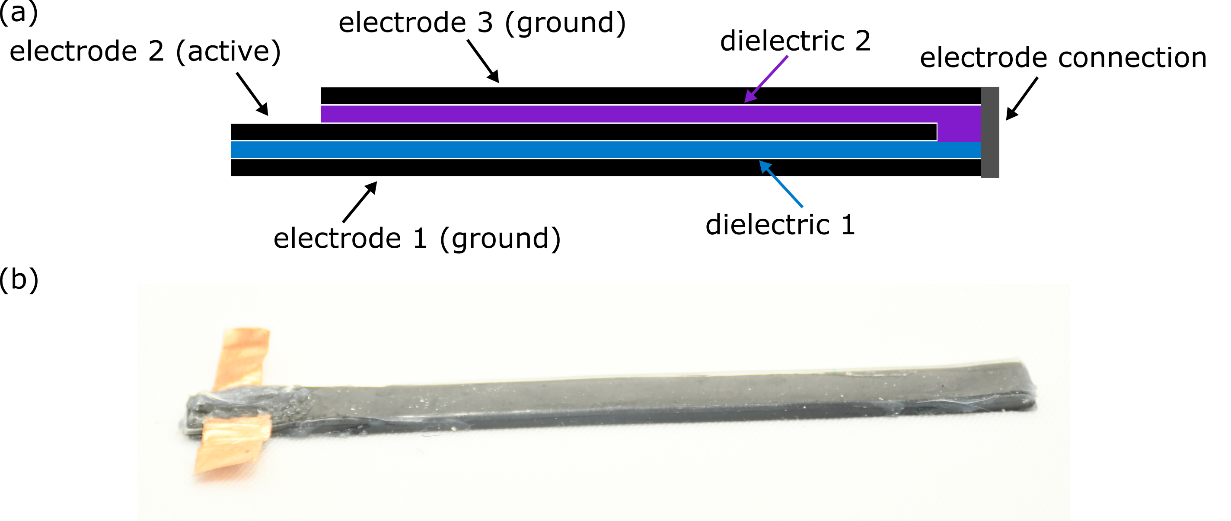


Figure S1: (a) Schematic of sensor layers and (b) image of sensor

Supplement: Supplemental data [file Suppl_FigureS1.docx]
