# Supplementary material for: Characterization of Temperature and Humidity Dependence in Soft Elastomer Behavior
Source: Soft Robot. 2024 Feb 13;11(1):118–30. doi: 10.1089/soro.2023.0004 (PMC10880277; doi:10.1089/soro.2023.0004)
Supplement: Supplemental data [file Suppl_FigureS3.docx]

#
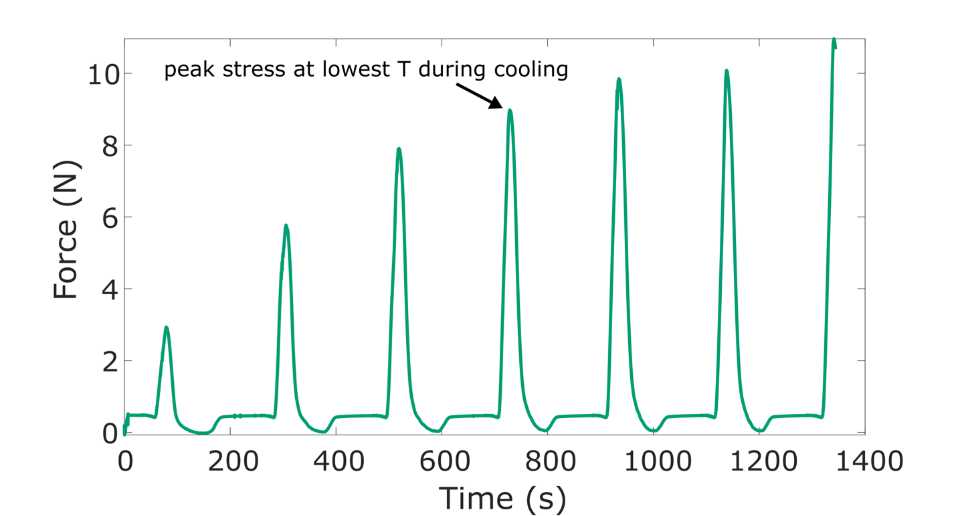
Stiffening behavior

Figure S3: Relaxation test of Dragon Skin sample, exhibiting extreme stiffening behavior.
