# Supplementary material for: Characterization of Temperature and Humidity Dependence in Soft Elastomer Behavior
Source: Soft Robot. 2024 Feb 13;11(1):118–30. doi: 10.1089/soro.2023.0004 (PMC10880277; doi:10.1089/soro.2023.0004)
Supplement: Supplemental data [file Suppl_FigureS4.docx]

#
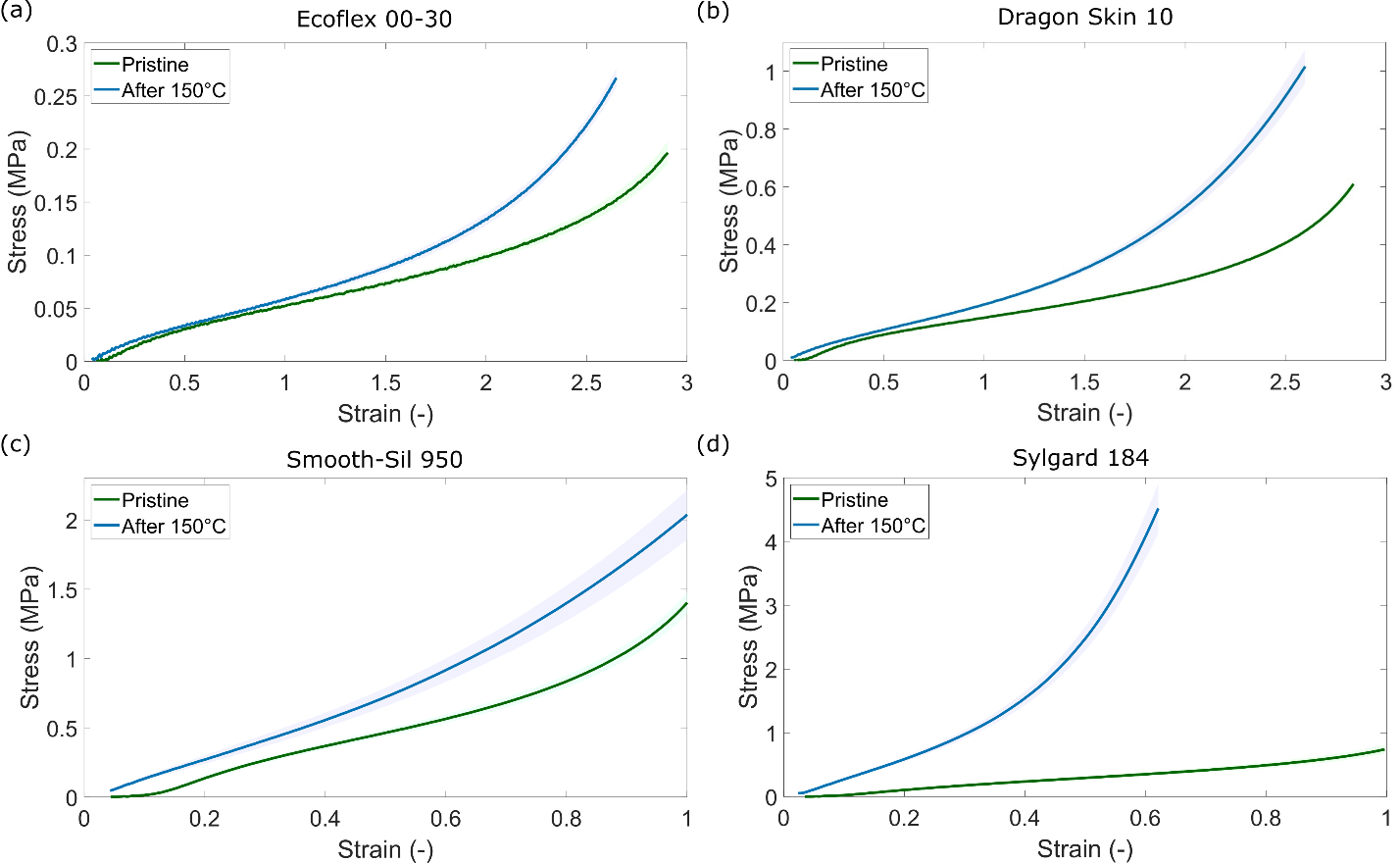
Thermal treatment

Figure S4: Stress-strain curves of samples prior to and post thermal treatment at 150° C for; (a) Ecoflex 00-30; (b) Dragon Skin 10; (c) Smooth-Sil 950; (d) Sylgard 184. Shaded areas represent standard deviations (n=6).
